# Effectiveness and Safety of Hypodermoclysis Patients With Cancer: A Single-Center Experience From Saudi Arabia

**DOI:** 10.7759/cureus.13785

**Published:** 2021-03-09

**Authors:** Sittelbenat Adem, Nabil ALMouaalamy

**Affiliations:** 1 Nursing, King Abdulaziz Medical City, Ministry of National Guard-Health Affairs, Jeddah, SAU; 2 Oncology Department/Palliative, Princess Noorah Oncology Center, King Abdulaziz Medical City, Ministry of National Guard-Health Affairs, Jeddah, SAU; 3 Research, King Abdullah International Medical Research Center, Jeddah, SAU; 4 College of Medicine, King Saud Bin Abdulaziz University for Health Sciences, Jeddah, SAU

**Keywords:** hypodermoclysis, hydration, palliative care, dehydration, community, esas-r scale

## Abstract

Introduction: Decreased intake of food or fluid causes dehydration in hospitalized adult patients. This has led to a negative impact on patients and increased the morbidity and mortality rate at the Princess Noorah Oncology Center, where patients with advanced cancer who suffer from dehydration have been treated with parenteral fluids until the date of discharge from the hospital.

Objective: The objective of this pilot study is to assess the effectiveness and safety of hypodermoclysis (HDC) to close the gap of treatment for home-based palliative patients with cancer.

Method: During home visits, the home health care (HHC) nurse assessed these patients through history and physical examination for dehydration. Our team also incorporated the Edmonton Symptom Assessment System revised (ESAS-r) Scale in the assessment of these patients' symptoms. Informed consent has been obtained from the patient and the caregiver. The trained nurses initiated the subcutaneous infusion. The caregivers monitored it and disconnected it when completed. The study population consists of palliative patients with advanced cancer under the services of palliative care. Effectiveness and safety have been assessed using the ESAS-r scale.

Results: A total of 25 (92.6%) HDC treatments were successfully completed for nine patients (seven males and two females). One female patient had only one session because her health had deteriorated for reasons other than dehydration and was transferred to the hospital. No serious side effects were observed. One (3.6%) patient developed redness at the site of cannula insertion. The mean duration of the infusions was 8.44 hours. The median age was 70 years.

Conclusion: This study has concluded that HDC is effective, safe, and can enhance the patient’s comfort level without the need for hospitalization. The fact that HDC can be administered at home with minimum equipment and technical support makes it an ideal option in several countries with varied income settings.

## Introduction

The World Health Organization (WHO) defines palliative care as an approach that concerns itself with the improvement of the quality of life of patients with life-threatening diseases. Palliative care aims to ameliorate patients’ physical symptoms, such as pain, and relieve suffering. However, treating physical symptoms is not the only element of palliative care. Palliative care also provides psychosocial treatment by encouraging patients to live actively and not passively wait for death and spiritual support [1].

In advanced cancer patients when oral intake is insufficient to maintain adequate hydration, most patients in traditional hospitals receive parenteral fluids [2,3]. In contrast, advanced cancer patients receiving home hospice care almost never receive parenteral fluid [4,5]. The peripheral intravenous (IV) route for hydration may be problematic for advanced cancer patients and hold a potential problem in the home care setting [6,7]. Specifically, disadvantages of the IV route for hydration in such patients include pain associated with needle insertion, the need for frequent site changes, difficulty in finding venous access, the need for immobilization of the arm, impediments to mobility, the risk of increasing agitation, and accidental catheter removal in patients with delirium, the need for hospitalization, high cost, and need for specific training in surveillance and care of complications, such as thrombophlebitis and infection [8].

Hypodermoclysis (HDC) is a technique of infusing fluids slowly into the subcutaneous tissue to hydrate older patients with poor venous access. Compared to IV, HDC costs less and has minimal discomfort and complications [9]. The fluid is absorbed into circulation via diffusion and perfusion [10,11]. Hypodermoclysis has a low incidence of adverse effects, generally related to local effects, such as swelling, localized pain, and erythema [12]. Other potential advantages of hypodermoclysis are that it is easy to manage in the home care setting and that the tubing can be connected and disconnected from the needle by primary caregivers after minimal training, it does not require expensive and complex infusion pumps, and it has the capability of intermittent administration. Hypodermoclysis is suitable for use in many hospital and homecare situations regardless of the patient’s age [13,14].

Based on the available evidence, HDC can benefit patients with dehydration in conjunction with poor venous access who are unable to take adequate food or fluid [15,16]. With this in mind, our team felt that starting those patients on HDC with the help of caregivers (after training) may relieve those symptoms and help them achieve their goal of staying home with their loved ones as long as possible without jeopardizing their safety. In a hospital setting, at the Princess Noorah Oncology Center (PNOC), advanced cancer patients receive IV infusion for nutrition, medications, or hydration. Upon discharge, an appointment is set for a follow-up at the palliative clinic with referral to home health care (HHC). This pilot study has been conducted to assess the utility, safety, and effectiveness of HDC hydration on palliative patients with dehydration.

The added advantage of this project is to help patients stay at home. HDC can reduce emergency room visits and hospital re-admissions [11,17]. Caregivers in our Saudi society associate hydration with compassion and as a means of nurturing their loved ones.

## Materials and methods

The study follows the observation method to assess home-based cancer patients who had been discharged from PNOC between January 2015 and February 15, 2016. The patients were referred to HHC and were seen within 14 days from the discharge date. On discharge, the patients were comfortable and had fair oral intake and no signs of dehydration. Although our team knows that it’s inevitable that those patients will get worse and will need more help later, we tried to achieve the patient and family wishes to be home with their loved ones. Before the initiation of the pilot project, all nurses were trained on how to start HDC and symptoms assessment before and after HDC. A 45-60 minute training session was given to all caregivers by the HHC palliative nurse on the administration of hypodermoclysis and infusion site assessment. In this study, we included adult patients with advanced cancer with dehydration (presented at home with decreased oral intake, lethargy, dry mouth, and scant urine output) in need of hydration and we excluded patients with hypovolemic shock and with no caregivers.

**Figure 1:**
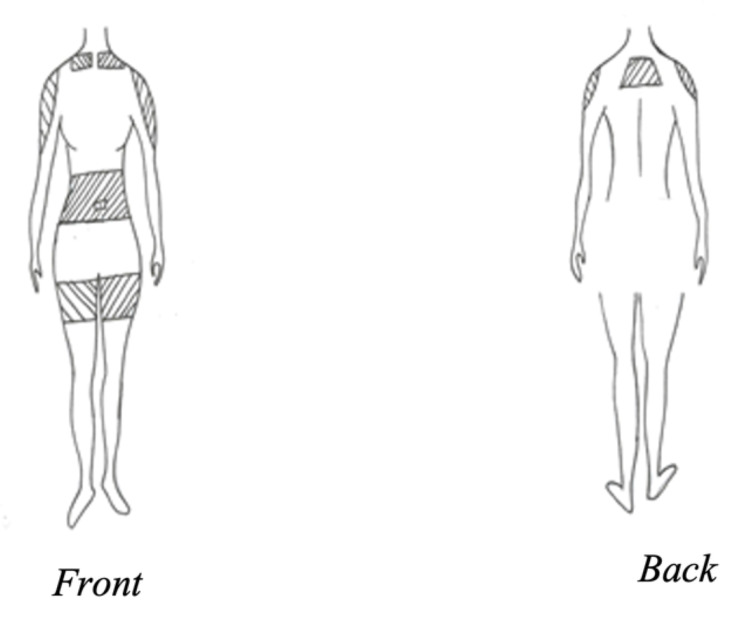
Site of Cannula Insertion (Shaded Areas)

During the home visits, the HHC nurse assessed these patients through history and physical examination for dehydration, also our team have incorporated the Edmonton Symptom Assessment System revised (ESAS-r) Scale in the assessment of these patients' symptoms and also to assess the improvements through ESAS-r score as well following the HDC infusion. The assessment findings were passed to the palliative consultant who ordered the HDC infusion electronically and faxed it and documents it in the patient medical records. The fluid type was chosen based on the palliative physician's decision which was based on baseline sodium level at discharge and also patient comorbidities (e.g. diabetes, hypertension, and heart failure). The HDC technique and nursing training is shown in Appendix A. The appropriate sites for cannula insertion, such as the upper back, chest, outer arms, abdomen, and outer thighs [18] are the shaded areas in Figure 1. The cannula was changed every 72-96 hours. The maximum volume delivered by gravity daily was 1000 ml. The rate of infusion was 0.5-2 ml per minute. Fluids infused per site were 1000 ml [19]. The duration of the infusion in a day was eight, 10, or 12 hours for one liter and half the time for 500 ml. In this study, the infusion fluid has no additives.

The HHC nurse obtained informed consent from them. The HHC nurse also calculated the rate based on the Manual of IV Therapeutics: Evidence-Based Practice for Infusion Therapy [20], prepared equipment, and initiated the infusion as in Appendix B.

The nurses start the infusion daily during the treatment period, and the family disconnects upon completion. The team focused on the identified issue using ESAS-r scale symptoms such as (pain, tiredness, nausea, depression, anxiety, drowsiness, appetite, feeling of well-being, and shortness of breath). The symptoms are categorized by severity: 0 (none), 1-3, (minimum) 4-6 (moderate), and 7-10 (severe) if the patient or family couldn't give a clear numerical value. The caregivers were contacted by phone every four hours. However, if they have any concerns, they were encouraged to contact the HHC nurse by phone.

The outcome of HDC was evaluated in terms of volume of fluid infused in 24 hours and improved dehydration symptoms that were present initially and also evaluated using the ESAS-r Scale before and after the treatment. The protocol used for applying HDC based on the hospital policy and procedures, Covenant Health Corporate Policy/Procedure #VII-B-110, and Full Scope of Practice 2014 [13]. Data collection included: demographic data, functional status, duration of the infusion (three days), type of solution, indication, adverse effects, family response, and outcome.

## Results

A total of 25 (92.6%) HDC treatments were successfully completed in nine advanced cancer patients. The patients were treated and followed up at home under the palliative care department at PNOC in Jeddah, Saudi Arabia. Most of the patients (77.8%) were males and the remainder were females. The mean age of the patients was equal to 71.89 years, SD=10.82 years, and most of them were married. Concerning their diagnosis, one patient had breast cancer, two patients had lung cancer, two had been diagnosed with colon cancer, one patient had gastric cancer, one patient had nasopharyngeal, another had hepatic, and one of them was diagnosed with prostate cancer. HDC treatments were successfully completed in nine patients (seven males and two females) (Table 1).

**Table 1:** Patients Sociodemographic and Disease-Related Characteristics. N=9.

	Frequency	Percentage
Sex		
Male	7	77.8
Female	2	22.2
Age ( years), mean (SD)		71.89 (10.82)
Marital state		
Married	8	88.9
Widowed	1	11.1
Diagnosis		
Breast cancer	1	11.1
Lung cancer	2	22.2
Colon cancer	2	22.2
Gastric cancer	1	11.1
Nasopharyngeal cancer	1	11.1
Hepatocellular cancer	1	11.1
Prostate cancer	1	11.1

All the patients had required HDC therapy at home, 33.3% of the patients received sodium chloride 0.9% solution, another 22.2% had received a sodium chloride 0.45% solution, but 11.1% had required HDC infusion of dextrose 5% with sodium chloride 0.9% solution, but also 22.2% had required HDC infusion of dextrose 5% with sodium chloride 0.45% solution, and 11.1% required a dextrose 3.3% with sodium chloride 0.3% solution. The mean duration of infusion days was equal to 3.22 days, SD=1.1 days, and the mean duration of the infusion in hours was equal to 8.44 hours, SD=2.74 hours. However, most of the patients (66.7%) had required a total of 1000 ml of the infused hypodermal fluid solution and 33.3% of the patients required a total of 500 ml of the hypodermal fluid solution. The family overall satisfaction with the therapy was measured, and most of the families were generally satisfied (Table 2).

**Table 2:** Descriptive analysis of the patients who received hypodermic fluid therapy.

Type of Intravenous Fluid therapy used	Frequency	Percentage
Sodium Chloride 0.9%	3	33.3
Sodium Chloride 0.45%	2	22.2
Dextrose 5% & Sodium Chloride 0.9%	1	11.1
Dextrose 5% & Sodium Chloride 0.45%	2	22.2
Dextrose 3.3% & Sodium Chloride 0.3%	1	11.1
Days of HDC fluids		3.22 (1.1)
Hours of HDC fluids transfusion		8.44 (2.74)
Amounts of IV fluid administered		
500 ml	3	33.3
1000 ml	6	66.7
Patients' family overall satisfaction		
Satisfied	8	88.9
Undecided	1	11.1

However the main outcome was measured on the patients themselves across three days, as the patients were asked to rate themselves for the extent of all ESAS-r Scale symptoms using an 0-10 Likert-like scale with a greater rating denoting the greater extent of the symptom. The most statistically and clinically significant symptoms during the assessment were nausea, loss of energy, and loss of appetite (Table 3).

**Table 3:** Descriptive analysis and non-parametric Friedman's test of the patients measured outcomes in response to hypodermic IVF therapy. N=9 Patients.

	Day-1/time1	Day-2/time2	Day-3/time3	test statistic	p-value
Nausea [0-10 Likert-scale mean (SD) rating]	5.56 (1.01)	4.00 (1.8)	2.67 (1.32)	χ2(2)=17	<0.001
Loss of appetite [0-10 Likert-scale mean (SD) rating]	4.78 (0.67)	3.22 (1.39)	2.00 (1.00)	χ2(2)=17.5	<0.001
Loss of energy [0-10 Likert-scale mean (SD) rating]	4.33 (1.12)	2.78 (1.40)	1.56 (0.88)	χ2(2)=15.10	0.001

**Figure 2:**
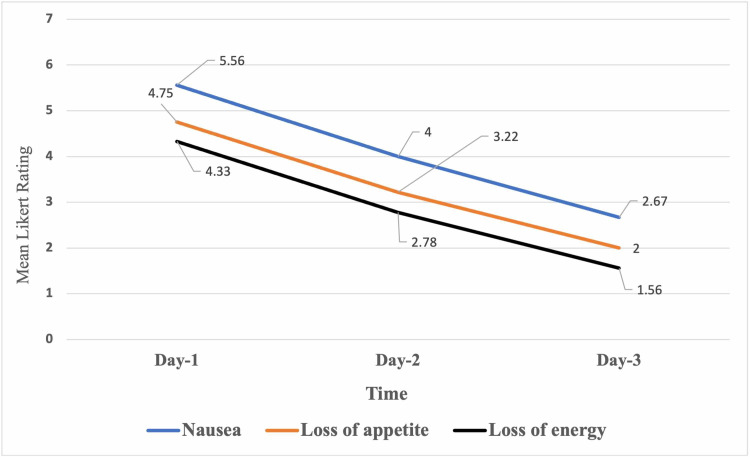
The patients mean self-rated extent of nausea, loss of appetite and energy across time during the hypodermic fluid therapy

A Friedman's non-parametric test was used to assess the statistical significance of the mean difference on patients' self-rating for the three symptoms (nausea, loss of appetite, and loss of energy) across the three time points during receiving the hypodermic fluid replacement therapy days. The resulted descriptive analysis ( means and standard deviations ) of patients' perception of nausea, loss of appetite, and energy are displayed in Table 3. The Friedman's test showed that there has been a statistically significant decline in patients feelings of nausea across time during the hypodermic fluid replacement period, χ2(2)=17, p<0.001, it is evident that the mean perceived nausea had declined from day one to day two then at day three, suggesting a linear declining trend that could be associated with the fluid replacement, also the patients mean self-rated sense of loss of appetite had declined significantly from baseline to the second day then to the third time point while receiving the HDC, χ2(2)=17.5, p<0.001, according to the Friendman's non-parametric test of paired repeated measures, likewise the patients self-rated mean perceived sense of loss of energy had declined significantly from day one to day two and day three, the mean perceived loss of energy had declined substantially between day one and day three, χ2(2)=15.10, p<0.001 according to the Friedman's test (Figure 2). The post-hoc pairwise comparisons for the three outcomes across the three time points suggested that only the third time points measured nausea, loss of appetite and energy differed significantly from the baseline measures for those patients perceptions, p<0.001 each respectively, the first and second day readings did not differ significantly for the three outcomes nonetheless. Furthermore, the patient's age did not correlate significantly with their perceptions of nausea, loss of appetite, and energy. However, the duration days had correlated significantly with the severity of nausea and loss of appetite and energy, r>0.50, p<0.050 each respectively well according to Pearson's test of correlation.

One patient (female) had one session but transferred to the hospital because her health deteriorated, not related to hydration. Regarding side effects, only one patient showed skin redness which subsided later with no intervention and was statistically insignificant.

## Discussion

This pilot study suggests that hypodermoclysis hydration is an acceptable choice for advanced cancer patients in Saudi Arabia. In addition, it shows that minimal training to the caregiver can result in self-satisfaction and help improving patient symptoms as well [21]. This study also shows that hypodermoclysis is a safe, effective, and useful hydration technique for palliative patients in our community [22,23].

Our results show that the infusion of an average of 833 ml was well tolerated over three to four days and made a significant impact on multiple symptoms, especially nausea, low energy, and low appetite, which was similar to what was found by Fainsinger et al. [5]. Our study also shows a hydration of an average of 833 ml over three to four days can help in hydrating advanced cancer patients and relieve some of their symptoms with minimal side effects and local toxicity, which correlates with the finding of a prospective open study of 100 consecutive patients in Canada, 69 of whom received subcutaneous hydration; the treatment was well tolerated in patients with an average volume of 1203 ± 505 ml/day although the infusion was continued for an average of 14 ± 18 days. 

The duration of our HDC was three to four days due to logistic issues especially manpower. Our study is the first to show that hydration for three to four days can help and relieve some symptoms, Several studies have shown that hydration for four to seven days was beneficial and some took 14-18 days [24].

In this study, we attributed the insignificance change in pain score over the treatment period to the fact that our patients were just discharged from the hospital (within 14 days) and the pain was well controlled. Family satisfaction with the intervention was remarkable, mainly because most of our patients and families in our community would like to stay home as much as they can and this study show clearly that hypodermoclysis can help achieve that goal. The families felt safe and requested it. Our experience taught us that both patients and caregivers will consider HDC again in the future. Our team recommended that attending nurses be vigilant in assessing dehydration. In this study, our team delivered subcutaneous fluid at a rate of 41 ml per hour. No pooling of fluid was noticed. The infusion time for 1000 ml was eight, 10, or 12 hours at a single site (500 ml in half the time). Following the HDC guidelines eliminates the potential adverse effects of subcutaneous hydration. Local massage eliminates mild subcutaneous edema. 

The duration of the infusion in a day was eight, 10, or 12 hours for 1 L and half the time for 500 ml. Since the solution runs slowly, no transient local edema was noticed. In this study, the infusion fluid has no additives.

Patient and family education and support promote safety and acceptance of subcutaneous hydration at home. This was emotionally beneficial for the patient and the caregiver. The Bioethics study showed an ethical dilemma when nutrition or fluid is withheld from palliative patients [25]. It is not surprising to see some families even demand fluid infusions at the end of life. However, the assessment has to be on an individual basis. In this study, the families expressed their satisfaction, showed the confidence to participate, and had a positive experience of the care. The families responded positively and requested the treatment, which continues for a few days more, as in Table 2.

We acknowledge that our study was limited because of a small sample of nine patients. There is a need for further studies in this area to validate outcomes with a larger sample.

Limitations

The sample size is small, which may decrease the statistical power, and is non-randomized. Furthermore, the published material on the topic is very limited. Only some observational or randomized controlled trial studies are available. Further study is needed, which will be designed as a randomized controlled trial to examine the efficacy of HDC infusion in providing drugs in a multicenter study.

## Conclusions

In this pilot project, our team implemented hypodermoclysis with considerable success in the home setting. Based on our results, we concluded hypodermoclysis is a safe and effective way to provide clinically indicated fluid volumes in adult palliative patients with dehydration and is well accepted by patients and families in Saudi Arabia. Also, minimal training of caregivers in our community can help the patient and the family achieve their goal of keeping the patient home as much as possible safely. HDC may serve as a perfect way to increase patient and caregiver satisfaction, decrease ER visits, and increase hospital revenue.

## Appendices

Appendix A

Training the nurses in-home health care

Training sessions for the nurses were prepared and delivered for a period of 60 minutes. The trainees practiced on a dummy first. They offered the infusion in the community under supervision.

Techniques of the Subcutaneous Infusion

Following the policy and procedure of the hospital and Covenant Health, we chose the proper sites for Cannula insertion. Choosing an insertion site depends on the available subcutaneous tissue and the state of the patient’s condition as indicated.

Areas to avoid are broken skin or irradiated, little subcutaneous, areas with lymphedema, infection, and ascites. 

Caregiver Training

The sessions for caregiver training ranged from 45 to 60 minutes. The home health care nurses hang the fluid, adjust the rate, and assess the site daily during the prescribed treatment period. The type of fluids ordered can be hypotonic, isotonic, or hypertonic, depending on the patient's condition.

Equipment

The choice of equipment is based on standard 64 of the Infusion Nurses Society's Infusion Nursing Standards of Practice.  

 Drip Rate Calculation                                                                                                                            

 The following formula used to calculate drip rate:

Volume x Drop factor   = drops per minute.

                                                      Time in minutes

For instance, if the ordered fluid is 1000 ml of 0.9% normal saline to be infused over 8 hours   

                       1000x20 = 42 drops per minute 

                                                                             8 x 60      

Appendix B

Initiation of Hypodermoclysis

1.      Assemble the equipment (fluid and tubing).

2.      Wash hands Don Gloves follow standard precautions and use aseptic technique.

The cannula insertion site is chosen based on adequate subcutaneous tissue, the patient's comfort, skin integrity, and patient's mobility.

3.      Swab the site with chlorhexidine in a circular motion, beginning at the center of the site.

4.      Insert the cannula with the needle, bevel up, in the subcutaneous tissue at a 45 to 60-degree angle, toward the head of the patient.

5.      Secure the needle and the tubing with an occlusive dressing (Tegaderm).

6.      Adjust fluid drip rate as prescribed to be infused via gravity.

The choice of equipment is based on standard 64 of the Infusion Nurses Society's Infusion Nursing Standards of Practice.

Equipment

Gloves 

1.      70% alcohol swab or chlorohexidine sticks

2.       Standard fluid infusion extension set with the drip chamber4 

3.      Solution bag (0.45% normal saline, 2/3 dextrose 1/3 normal saline infusion, 0.9% normal a saline and dextrose 5%)

4.      70% alcohol swab or chlorohexidine sticks

5.      Cannula 24 or 25 gauge

6.      Semipermeable transparent dressing for insertion site e.g., Tegaderm

7.      Drip stand (optional at home can use dress hanger) and 

8.      Sharps box 

9.      Sterile cap,

10.    Gauze 2x2 cm

11.    Sharps box.
